# A Novel Clinical Grading Scale to Guide the Management of Crusted Scabies

**DOI:** 10.1371/journal.pntd.0002387

**Published:** 2013-09-12

**Authors:** Joshua S. Davis, Steven McGloughlin, Steven Y. C. Tong, Shelley F. Walton, Bart J. Currie

**Affiliations:** 1 Global and Tropical Health Division, Menzies School of Health Research, Darwin, Northern Territory, Australia; 2 Department of Infectious Diseases, Royal Darwin Hospital, Darwin, Northern Territory, Australia; 3 Intensive Care Unit, The Alfred Hospital, Melbourne, Victoria, Australia; 4 Inflammation and Healing Research Cluster, Faculty of Science, Health, Education and Engineering, University of the Sunshine Coast, Maroochydore, Queensland, Australia; University of California San Diego School of Medicine, United States of America

## Abstract

**Background:**

Crusted scabies, or hyperinfestation with *Sarcoptes scabiei*, occurs in people with an inadequate immune response to the mite. In recent decades, data have emerged suggesting that treatment of crusted scabies with oral ivermectin combined with topical agents leads to lower mortality, but there are no generally accepted tools for describing disease severity. Here, we describe a clinical grading scale for crusted scabies and its utility in real world practice.

**Methodology/Principal Findings:**

In 2002, Royal Darwin Hospital (RDH), a hospital in tropical Australia developed and began using a clinical grading scale to guide the treatment of crusted scabies. We conducted a retrospective observational study including all episodes of admission to RDH for crusted scabies during the period October 2002–December 2010 inclusive. Patients who were managed according to the grading scale were compared with those in whom the scale was not used at the time of admission but was calculated retrospectively. There were 49 admissions in 30 patients during the study period, of which 49 (100%) were in Indigenous Australians, 29 (59%) were male and the median age was 44.1 years. According to the grading scale, 8 (16%) episodes were mild, 24 (49%) were moderate, and 17 (35%) were severe. Readmission within the study period was significantly more likely with increasing disease severity, with an odds ratio (95% CI) of 12.8 (1.3–130) for severe disease compared with mild. The patients managed according to the grading scale (29 episodes) did not differ from those who were not (20 episodes), but they received fewer doses of ivermectin and had a shorter length of stay (11 vs. 16 days, p = 0.02). Despite this the outcomes were no different, with no deaths in either group and a similar readmission rate.

**Conclusions/Significance:**

Our grading scale is a useful tool for the assessment and management of crusted scabies.

## Author Summary

Crusted scabies is a severe skin condition caused by a microscopic parasitic mite. It occurs in people whose immune system does not react properly to the mite and it leads to crusting and cracking of the skin and can cause death. The usual treatment for crusted scabies is a tablet called ivermectin combined with anti-scabies skin creams. However, there is no current method of measuring the severity of crusted scabies and thus deciding how long to continue the treatment for. We have developed a grading scale based on examination of the skin, which classifies patients as mild, moderate or severe, and uses this grading to suggest the duration of treatment. We have trialed this grading scale over an 8-year period in 49 episodes of crusted scabies requiring hospital admission, and have found that it leads to a shorter length of hospital stay and treatment, but equivalent outcomes compared to those who were treated without the use of the grading scale.

## Introduction

Scabies is a parasitic infestation caused by the mite *Sacroptes scabiei var hominis*. Globally, over 300 million people are estimated to be affected [1]. The mite is endemic in disadvantaged and impoverished communities [2], [3]. In Australia Indigenous people suffer a significant disadvantage in health outcomes compared with non-Indigenous Australians [4], [5], and scabies is endemic in many Indigenous communities in northern Australia, with a recent survey demonstrating a mean prevalence of 13.4% in five remote Indigenous communities [6].

Crusted scabies (also known as “Norwegian scabies”) is hyperinfestation with the *Sarcoptes scabiei* mite, and is characterized by a non-protective host immune response, the development of hyperkeratotic skin crusts and skin fissuring [7]. It is a severe disease with a significantly higher mortality than ordinary scabies. Unlike ordinary scabies, where there are usually less than 20 mites on the host's entire skin, individuals with crusted scabies can have up to 4000 mites per gram of skin and are extremely infectious to others [8], [9]. Despite the severity of the disease there is significant variability in the clinical presentation, and there is currently no generally accepted method of describing the severity of a crusted scabies infection.

The optimal treatment for crusted scabies has not been subjected to a comparative trial and is generally based on expert opinion [10], [11]. However observational data suggest that the use of multiple doses of oral ivermectin as therapy for crusted scabies can lead to a significant decline in mortality [9], [12], [13]. In an attempt to formalize and improve the treatment of crusted scabies, we developed a grading scale, based on our clinical experience in managing such patients. This was introduced into routine clinical use at our hospital in 2002, and has been used since this time to titrate the duration of ivermectin and topical therapy to illness severity.

Here, we describe the grading scale and our experience with it over the first eight years of its use. We aimed to evaluate the utility of the grading scale, including its correlation with other putative markers of illness severity, the safety of its use and the effect on length of stay and relapse rates.

## Methods

### Ethics statement

The study was approved by the human research ethics committee of the Menzies School of Health Research and Northern Territory Department of Health.

### Study setting

350 bed tertiary referral hospital in the tropical Northern Territory, Australia, serving a population of approximately 150,000 people spread over an area of 500,000 km^2^, including many remote Indigenous communities. Local policies encourage the hospitalization of patients with crusted scabies for clinical management, as well as environmental health input to address the risk of ongoing transmission in an index patient's household. The standard treatment protocol for crusted scabies includes prolonged hospitalization in a single room with contact precautions, the use of topical benzyl benzoate plus 5% tea tree oil 2–3 times per week [14], multiple doses of oral ivermectin (as described below), topical keratolytics, systemic antibacterial drugs where judged clinically necessary, and attention to medical comorbidities.

### Participants

All patients admitted to our hospital with a discharge diagnosis of crusted scabies between 1^st^ of October 2002 and 31^st^ of December 2010 were included in the study. Crusted scabies was diagnosed based on the clinical opinion of an Infectious Diseases specialist, supplemented by skin scrapings demonstrating *S. scabiei* mites on microscopy.

### Severity grading scale

**Figure 1:**
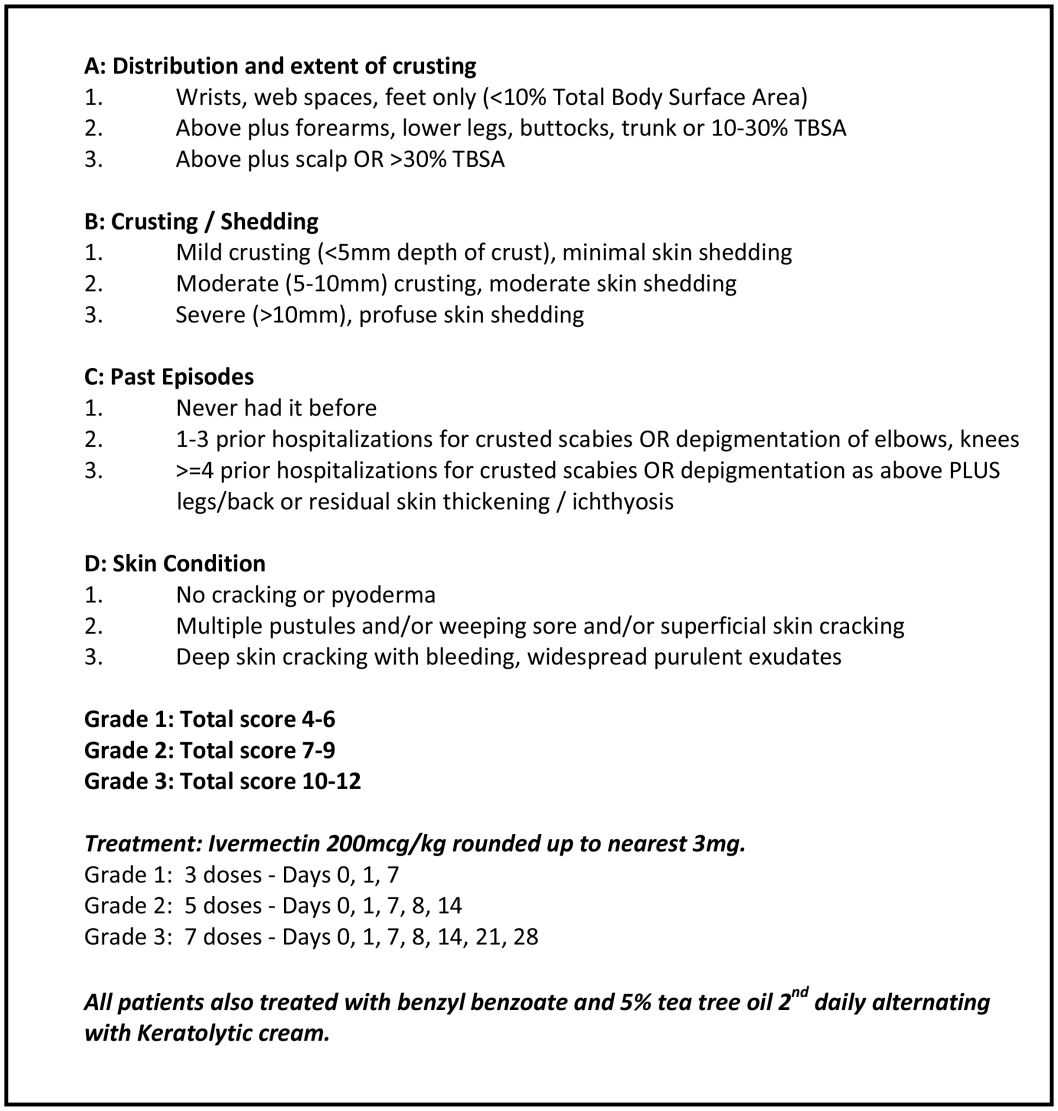
Severity grading scale for crusted scabies.

The grading scale for crusted scabies is shown in **Figure 1**. It is based on clinical assessment in four key areas: the distribution and extent of crusting; the depth of crusting; the degree of skin cracking and pyoderma; and the number of previous episodes. This scale was developed in 2002 by two of the authors (JD and BC) for use with all patients hospitalised with crusted scabies. It was partly based on previous local experience that multiple doses of ivermectin in addition to topical treatment were more effective than topical treatment alone for the treatment of crusted scabies [9]. Other studies have confirmed the efficacy of the combination of ivermectin and topical therapy for crusted scabies [13], [15], [16]. During the study period medical staff managing patients with crusted scabies were encouraged but not compelled to use the grading scale to guide management. Therefore we were able to compare those patients in whom the grading scale was applied at the time of the patient's clinical presentation to those in whom the grading scale was not used and then calculated retrospectively by the authors.

### Data definitions, collection and analysis

We reviewed clinical notes, bedside charts and the hospital's clinical pathology database for each patient using a standardized case record form. We collected data on demographics, comorbidities, disease severity, grading scale and outcomes. Where the grading scale had not been prospectively documented, we calculated it based on the detailed clinical information found in the medical record. Each admission (rather than each individual patient) was counted as a discrete episode. Where a patient had more than one admission during the study period, it was only counted as a separate episode if at least 30 days had elapsed from the previous date of discharge. Iatrogenic immunosuppresion was defined as the use of any of the following medications within the past 3 months: prednisolone ≥0.5 mg/kg/day or equivalent for at least 14 days; immunosuppresion for solid organ transplant; cancer chemotherapy; immunosuppressive monoclonal antibody use; any other use of azathioprine, methotrexate, leflunomide, cyclosporine, mycophenolate, or cyclophosphamide. Hazardous alcohol use was defined as an average of >4 standard drinks per day for a man or >2 for a woman. Chronic renal disease was defined as an estimated glomerular filtration rate of less than 30 ml/min, or the need for dialysis.

Data were entered into a purpose-built database using Epidata v 3.0 and were analysed using Stata version 10 (Statacorp, College Station, Texas, USA). Categorical variables were compared using Fisher's exact test, and continuous using Mann-Whitney-U test. Correlations were assessed using Spearman's rank correlation. P values of <0.05 were considered significant

## Results

### Demographics and comorbidities

There were 49 admissions for crusted scabies in 30 patients during the eight year study period. Of the episodes, 49 (100%) were in Indigenous Australians, 29 (59%) were male and the median age at the time of the first admission within the study period was 45.4 years (**Table 1**). Most of the patients lived in remote areas, and iatrogenic immunosuppresion was rare.

**Table 1:** Demographics and comorbidities.

	n	%
**Indigenous**	49	100
**Male**	29	59
**Age (years)** a	44.1 [34.6–53.5]	-
**Remote-dwelling**	39	80
**Diabetes**	11	22
**Chronic Renal Disease**	8	16
**Chronic liver disease**	6	12
**Iatrogenic immunosuppression**	3	6
**Hazardous alcohol use**	24	48
**HTLV-1 infection**	5	10
**HIV infection**	0	0

a Median [IQR].

### Patient management

All patients received at least one dose of oral ivermectin (with a mean of 5.2 doses, and a range of 2 to 10). 47 patients (95%) were treated with topical benzyl-benzoate in combination with 5% tea-tree oil, and the remainder with topical permethrin. In addition, all patients were treated with topical Calmurid (lactic acid and urea in sorbolene cream, used as a keratolytic). Systemic antibiotics were used in 38 (79%) of episodes.

### Disease severity and outcomes

According to the grading scale, 8 (16%) episodes were mild (grade 1), 24 (49%) were moderate (grade 2), and 17 (35%) were severe (grade 3). Seven episodes (14%) were complicated by bacteraemia, with the causative organism being *Staphylococcus aureus* in 6 patients, and a mixed infection with Group A streptococcus and *Escherischia coli* in 1. The disease severity according to the grading scale did not correlate with the proportion of patients with bacteremia, or with the peak plasma C-reactive protein during the admission (table 2). However, there was a non-significant trend towards lower nadir plasma albumin and longer hospital stay with higher severity (**table 2**). No patients in this cohort died during the hospital admission, but a substantial proportion (47%) required readmission for crusted scabies within the eight year study period. Readmission was significantly more likely with increasing disease severity, with an odds ratio (95% CI) of 5.9 (0.7–55.9) for moderate disease compared with mild, and 12.8 (1.3–130) for severe disease compared with mild.

**Table 2:** Grading scale, disease severity, and outcomes.

	Mild	Moderate	Severe	Total	P^d^
**n**	8	24	17	49	
**Bacteraemic** a	1 (13%)	4 (17%)	2 (12%)	7	NS
**Peak CRP** b **(units)**	46 [28-145]	31 [6-58]	49 (29-54)	-	NS
**Nadir albumin (g/dL)** c	33 [27-36]	31 [22.5-34.5]	26 (23-31)	-	NS
**ICU admission** a	1 (13%)	2 (8%)	2 (12%)	5	NS
**Required readmission** d	1 (13%)	11 (46%)	11 (65%)	23	0.03
**Length of stay (days)** e ^,^ f	13 [6-21]	14 [11-17]	15 [13-16)		NS
**Doses of ivermectin** e ^,^ f	3 [3-3]	5 [5-5]	7 [7-7]		0.001

a n(%).

b Highest plasma C-reactive protein during the hospital admission (median [IQR]).

c Lowest serum albumin value during the hospital admission (median [IQR]).

d P value for moderate and severe combined, compared with mild, except where indicated.

e Median [IQR].

f P value based on Kruskall Wallis test comparing the three groups.

### Effect of prospective use of the grading scale

There was no significant difference in age, gender, location of residence or comorbidities between those patients who had the severity score calculated at the time of admission (n = 29) and those who did not (n = 20). Episodes where the grading scale was calculated at the time of admission had a significantly shorter length of stay, and received fewer doses of ivermectin than those not managed using the grading scale (**Table 3**). Despite this their outcomes were no different, with no deaths in either group, and a similar readmission rate in the two groups.

**Table 3:** Relationship between prospective use of the grading scale and clinical outcomes.

	Grading scale used	Grading scale not used	P value
**Number of episodes**	29	20	-
**Mild** a	4 (14%)	4 (20%)	NS
**Moderate** a	12 (41%)	12 (60%)	NS
**Severe** a	13 (45%)	4 (20%)	NS
**Length of stay (days)** b	11 [9–16]	16 [13–21]	0.02
**Number of doses of ivermectin** c	4.7 [2]	5.5 [1.5]	NS
**Hospital mortality**	0	0	-
**Readmission required** d	13 (45%)	10 (50%)	NS

a Disease severity according to grading scale; n(%).

b Median [IQR].

c Mean [sd].

d Readmission for crusted scabies within the 8 year study period, n(%).

## Discussion

This is the first published description of a clinical severity grading scale for use in patients with crusted scabies. The use of this grading scale in our setting is associated with good outcomes despite shorter hospital stays and less ivermectin use compared with those managed without the use of the grading scale.

Crusted scabies is a severe disease with significant morbidity and mortality which is more prevalent in communities such as remote-dwelling Australian Indigenous people [2]. Crusted scabies is usually reported as occurring in patients who are immunosuppressed, either iatrogenically [17], [18], [19], [20] or by retroviral infection [21], [22], [23]. In our cohort there was a high rate of hazardous alcohol use, diabetes and chronic renal disease, but only 16% of episodes were associated with iatrogenic immunosuppresion or HTLV-1 infection. This reinforces the findings of previous studies that, in Indigenous Australians, the majority of people with crusted scabies do not meet the generally accepted definitions of significant immunosuppression and suggests that the immune defect in patients with crusted scabies is subtle and probably multifactorial [8].

Our grading scale did not correlate with many of the putative measures of disease severity we used (CRP, ICU admission, bacteraemia). However, these factors are really measures of the sequelae of crusted scabies and there is no generally accepted single marker of disease severity in this setting (hence the need for the clinical grading scale). The degree of systemic inflammation and risk of bacteraemia are likely to relate to multiple factors, including the patient's immune responses, the depth of skin cracks, the degree of bacterial skin colonization and the patient's underlying comorbidities. Hence this lack of correlation does not necessarily imply that the grading scale does not reflect disease severity.

Long hospital stays (particularly those involving single rooms and contact isolation) are expensive to the health care system, and frustrating for patients. The 5 day decrease in length of stay which we observed with the use of the grading scale, with no increase in relapse rates, is substantial and represents a large cost saving. Another potential advantage of our grading scale is that it may help guide the duration and type of therapy for those clinicians who are less experienced in the management of crusted scabies. Given that crusted scabies is a rare condition in most settings, the utility of such a grading scale for the average clinician is a good reason for its use.

Ivermectin is an orally administered semi-synthetic macrocyclic lactone antibiotic. It is approved for the treatment of scabies in France but is not licensed for the treatment of scabies in the United States, United Kingdom or Australia. However is it commonly used off-label for the treatment of scabies in Australia. Ivermectin does not sterilize scabies eggs so multiple doses are recommended to kill newly hatched mites [10]. Ivermectin has been associated with adverse effects in some studies, which emphasizes the benefit of using a grading scale that allows for the titration of the total dose of ivermectin and in our study a possible reduction in number of doses in patients with milder disease. Intensive ivermectin use may also increase the probability of the mite developing resistance especially in patients with multiple relapses [24].

This study was planned prospectively, but the grading scale had to be calculated retrospectively in 40% of patients, introducing possible inaccuracies in the calculated scores. Fortunately, a detailed clinical assessment was recorded in the medical record for all patients, and thus we were able to calculate the score for all patients without having to interpolate missing data. Despite this, the score calculated at the time of clinical assessment is likely to be more accurate; retrospectively calculated scores may have underestimated severity in certain areas such as degree of crusting and shedding. However, this would not affect the overall conclusions regarding the use of the score, as the outcome measures were not the scores themselves, but objective measures including length of hospital stay and need for re-admission. Our population differs substantially from some others in whom crusted scabies has been reported to occur. Hence it is important for the grading scale to be studied in other populations before concluding that it is useful in all settings.

We have described a simple clinical grading scale to aid in the management of patients with crusted scabies. If validated in other settings, its use is likely to improve the management of crusted scabies and may lead to a decreased length of required hospital stay and of ivermectin treatment, without compromising outcomes.

## Supporting Information

Checklist S1 STROBE checklist.
(DOC)
